# miR-10b suppresses cell invasion and metastasis through targeting HOXA3 regulated by FAK/YAP signaling pathway in clear-cell renal cell carcinoma

**DOI:** 10.1186/s12882-019-1322-1

**Published:** 2019-04-11

**Authors:** Cheng He, Zhi-Yong Chen, Yang Li, Zhong-Qing Yang, Feng Zeng, Yu Cui, Yao He, Jin-Bo Chen, He-Qun Chen

**Affiliations:** Department of Urology, Xiangya Hospital, Central South University, No. 87, Xiangya Road, Changsha, 410000 Hunan Province People’s Republic of China

**Keywords:** miR-10b, ccRCC, Metastasis, HOXA3, FAK/YAP

## Abstract

**Background:**

MicroRNAs have been related to tumor progression in diverse human cancers including clear-cell renal cell carcinoma (ccRCC). Previous study has suggested the important regulation function of miR-10b in ccRCC. However, the direct target of miR-10b in ccRCC and the related molecular mechanisms has not yet been revealed.

**Methods:**

miR-10b and HOXA3 was detected by qRT-PCR. MTT, colony formation assay, wound-healing and transwell assays were performed to detect cell proliferation, colony formation, migration, and invasion abilities in ccRCC. Western blot analyses were performed to evaluate the protein expression of HOXA3, YAP, FAK and MMP-9. Dual luciferase reporter assay was employed to measure potential molecular mechanism of miR-10b in ccRCC.

**Results:**

miR-10b was down-regulated in 786-O and A498 cells as compared to renal tubular HK-2 cells. By contrast, HOXA3 and YAP was up-regulated in ccRCC cells and tissues. Functionally, knockdown of YAP inhibited cell proliferation, migration and invasion. Knockdown of FAK downregulated YAP, in turn, resulted in a decrease of HOXA3 expression. Mechanically, miR-10b targets HOXA3 to exert its tumor-suppressive effect on ccRCC in vitro.

**Conclusions:**

These novel data suggest that miR-10b suppresses cell invasion and metastasis through targeting HOXA3, which partially passed through the FAK/YAP signaling pathway.

## Introduction

Clear cell renal cell carcinoma (ccRCC) is the most common type of RCC, responsible for approximately 75–80% of cases. It is the second leading cause of death from urologic malignancies, which is characterized by extraordinarily high rates of local invasion, malignancy, and mortality, and resistance to chemotherapy and radiotherapy [1–4]. When diagnosed, around 25–30% of patients present with metastatic disease [5]. Although ccRCC treatment has achieved substantial advance in recent years [6, 7], most treated patients eventually develop progressive disease owing to acquired resistance [8, 9]. Hence, disclosing the molecular mechanisms underlain will offer promise for ccRCC treatment.

microRNA-10b (miR-10b) has been suggested to be dys-regulated in a number of cancers and to act as a key regulator of cell invasion and metastasis [10]. It is usually viewed as an oncomiR that regulates tumor suppressors and is up-regulated in breast cancer with distant metastasis, esophageal, pancreatic, and bladder cancers [11–14]. By contrast, several studies revealed that miR-10b is down-regulated in RCC and is inversely associated with patient survival [15–18]. The mechanism for down-regulation of miR-10b in ccRCC, however, remains unknown.

Homeobox (HOX) protein has been recognized as key determinants of cell identify and potential targets during tumorigenesis [19]. HOXA3, the HOXA gene near the 3′ end of the cluster was found to induce cell migration in endothelial and epithelial cells [20] possibly through cancer-associated hypermethylation [21]. Previous studies have suggested that HOXB3 functions as a tumor suppressor in RCC [22] and that HOXA3 is a potential target of miR-10b in cell proliferation [23]. The HOXA3 in the regulation of RCC is thus warrant further investigation.

Yes-associated protein (YAP), the effector of the Hippo tumor-suppressor pathway that plays a critical role in stem cell proliferation and organ size control, has been identified a potential oncogene in multiple cancers [24–26]. YAP regulates the expressions of HOXA3 in oral and dental epithelial tissues and in the epidermis of skin during embryonic and adult stages [27]. This thus provides insight into the molecular mechanisms linking abnormal YAP activities in human ccRCC.

Focal adhesion kinase (FAK) is a key molecule in focal adhesions and regulates cell growth, survival, and migration. It is a pivotal mediator of cell signaling, and relays external mechanical stimuli to other transducers, YAP being one of the core ones, within the cytoplasm. Downstream effects of FAK activation involve cell survival, proliferation, and motility, and therefore FAK represents a potential target for cancer therapy [28].

In the current study, we characterized miR-10b and HOXA3 expression in ccRCC cells and evaluated the influence of manipulating YAP and FAK expression on HOXA3 expression in vitro. We demonstrated that miR-10b, through targeting HOXA3 regulated by FAK/YAP signaling pathway, suppresses cell invasion and metastasis of ccRCC.

## Materials and methods

### Human clinical samples

Six paired human ccRCC tissues and corresponding non-tumor control tissues were obtained from Xiangya Hospital of Central South University. This study gained approval from the Ethics Committee of Xiangya Hospital of Central South University, and consents from patients who provided the clinical samples. The clinical information and pathological characteristics of the 6 patients with ccRCC are presented in Table 1.

**Table 1:** Clinical characteristics of 6 patients with ccRCC

Patient	Gender	Age (years)	Cancer type	Tumor size (cm)	Grade	Stage
1	Female	50~60	I	5 × 4 × 3	Well-differentiated	T_1b_N_0_M_0_
2	Male	60~70	III	4 × 3 × 3	Moderately differentiated	T_3a_N_0_M_0_
3	Female	60~70	I	6 × 4 × 2	Moderately differentiated	T_1b_N_0_M_0_
4	Male	60~70	I	2 × 1 × 1	Poorly differentiated	T_1a_N_0_M_0_
5	Female	60~70	I	5 × 5 × 5	Well-differentiated	T_1b_N_0_M_0_
6	Male	50_60	II	6 × 6 × 6	Moderately differentiated	T_2_N_0_M_0_

### Cell lines and culture conditions

The primary non-metastasis human ccRCC 786-O, and A498 cells, renal tubular HK-2 cells, and non- von Hippel-Lindau (VHL) mutated cancer CAKI cells were purchased from the American Type Culture Collection (ATCC) (Rockville, MD, USA). Cells were cultured in DMEM medium supplemented with 10% fetal bovine serum (FBS) with 1% antibiotics and maintained at 37 °C supplied with 5% CO_2_ humidified atmosphere.

### RNA extraction and quantitative reverse transcription PCR (qRT-PCR)

Total RNA was isolated with TRIzol Reagent (Invitrogen™, China). Complementary DNA was synthesized with random primers using a reverse transcription kid PrimeScript RT reagent kit (Takara Biomedical Technology, Dalian, China). Quantitative real-time PCR (qPCR) analysis was performed using the StepOnePlus Real-Time PCR System (Applied Biosystems, Foster City, CA, USA). The primer sequences were as follows: miR-10b: 5′-TACCCTGTAGAACCGAATTTG-3′ (forward) and 5′-AACTGGTGTCGTGGAGTCGGC-3′ (reverse); HOXA3: 5′-AAGGTCTAGAGGTTG CTGGAATGGCTGTAT-3′ (forward) and 5′-AAGGTCTAGAGGTGACTCTCCCCA GTTCAG-3′ (reverse); YAP: 5′-GAACCCCAGATGACTTCCTG-3′ (forward) and 5′-CTCCTTCCAGTGTTCCAAGG-3′(reverse); FAK, 5′- GCGGCCCAGGTTTACTGAA-3′ (forward) and 5′- GGCCTGTCTTCTGGACTCCA-3′(reverse); MMP-9, 5′-CCTGGAGACCTGAGAACCAATCT-3′(forward) and 5′-CCACCCGAGTGTAACCATAGC-3′(reverse); β-actin, 5′-CTACGTCGCCCTGGACTTCGAGC-3′ (forward) and 5′-GATGGAGCCGCCGATCCACACGG-3′(reverse); and RNU44, 5′-CCTGGATGATGATAGCAAATGC-3′(forward) and 5′- GAGCTAATTAAGACCTTCATGTT-3′(reverse). β-actin was used as the endogenous control, while RNU44 was used as internal control for miR-10b, and the ΔΔCt method for relative quantification of gene expression was used to determine mRNA expression levels.

### Western blotting analysis

Cultured cells were harvested and lysed in ice-cold radio immunoprecipitation assay buffer (RIPA, Beyotime, Beijing, China) supplied with 0.001% protease inhibitor cocktail (Roche, Pleasanton, CA, USA) and incubated on ice for 30 min. BCA Protein assay kit (Beyotime) was then used to detected the concentrations of protein samples. Equal amounts of protein extracts were run on a 10% SDS-PAGE gels and transferred eletrophoretically to polyvinylidene fluoride membranes (Milipore, Shanghai, China). The membranes were blocked with 5% nonfat milk, followed by incubation with primary antibodies at 37 °C overnight. Primary antibodies used were antibodies specific against HOXA3 (1:200, Sigma-Aldrich), YAP (1:500, Sigma-Aldrich), MMP-9 (1:500, Sigma-Aldrich), MMP-2 (1:500, ab37150, Abcam), FAK (1:500, Sigma-Aldrich), and Slug-1 (1:500, ab106077, Abcam). Membranes were the incubated with anti-rabbit secondary antibody (Sigma-Aldrich) and visualized by densitometry using Quantity One software (Bio-Rad) with β-actin as a control.

### MTT assay

Cell viability was measured using the 3-(4,5-dimethylthiazol-2-yl)-2,5-diphenyltetrazolium bromide (MTT) assay (Sigma Aldrich). In brief, 786-O and A-498 cells (~ 2 × 10^4^) were seeded into 96-well plates overnight. After 72 h of transfection, 20 μl MTT was added into each well, and cells were incubated for 4 h at 37°C. Next, 150 μl dimethyl sulfoxide (DMSO) was added to solubilize the precipitate. The optical absorbance of each sample was measured at 490 nm using PowerWave XS machine (BioTek, Vermont, USA).

### Colony formation assay

Cells were seeded into a six-well culture plate and transfected with miR-10b or NC for 24 h. A total of 1 × 10^2^ cells were then incubated for at 37°C for 2 weeks. Cell colonies were washed twice with PBS and subsequently stained with Giemsa dye. Colonies (≥ 50 cells as a colony) were examined using a surgical microscope, with the efficiency of colony formation expressing as (number of colonies/number of cells inoculated) × 100%.

### Wound healing assay

For wound healing assay, transfected cells were seeded into six-well plates and cultured overnight. Wounds were made with a sterile 200 μl plastic pipette tips to scratch cell layer and washed with culture medium. Cells were then incubated for 24 h in serum-free DMEM-medium with 1% FBS. Images were obtained under a microscopy (Olympus, Tokyo, Japan) at different time points at 100× magnification.

### Transwell assay

For transwell invasion assays, cells were placed into 24-well transwell chambers with the upper chambers coated with 150 μg of Matrigel (BD Bioscience, Bedford, MD, USA). The chambers were then incubated for 24 h in 500 μl DMEM-medium containing 10% FBS before examination. The cells remaining on the upper membrane surface were removed, whereas the cells adhering to the lower membrane surface were fixed, stained in a dye solution with 0.5% crystal violet and counted in five random fields at 200 × magnification. For transwell migration assays, cells were placed into 24-well transwell chambers with the upper chambers coated without Matrigel.

### Lentivirus infection and oligonucleotide transfection

The miR-10b lentivirus was obtained from GeneChem (Shanghai, China). The constructs contained the pre-miR10b sequences and 100 bases of upstream and downstream flanking these sequences and were cloned into the pGCSIL-GFP vector. The 786-O and A498 cells (1 × 10^5^) were infected with 1 × 10^7^ lentivirus transducing units in the presence of 10 μg/ml polybrene, with the empty lentiviral vector used as negative control. The miR-10b inhibitor, mimic, negative control for mimic, negative control for inhibitor, negative control for shRNA and shRNA of YAP, HOXA3 were designed and synthesized by RiboBio. Target cells were transfected using Opti-MEM and Lipofectamine 3000 reagents (Invitrogen; Therom Fisher Scientific, Inc.).

### Luciferase reporter assay

To construct a luciferase reporter vector, the RNA sequence of wide-type HOXA3 (HOXA3-WT) was PCR-amplified and inserted into the pGL3 luciferase promoter plasmid (Promega Corporation, Madison, WI, USA). HOXA3 RNA sequence with mutations in the putative binding site (HOXA 3′-MUT) was also inserted into the pGL3 luciferase promoter plasmid and was chemically synthesized by Shanghai GenePharm Co., Ltd. Target cells were seeded in 96-well plates and reporter plasmids were co-transfected with either miR-10b mimics or a negative control using the Lipofectamin 3000 method. After 48 h of transfection, cells were lysed and the Firefly and Renilla luciferase activities were examined using the Dual-Luciferase Reporter Assay System (Promega).

### Statistical analysis

Data were expressed as mean ± SD. The statistical difference among different groups was analyzed by Student’s t-test and one-way ANOVA using SPSS 22.0 software (SPSS Inc., Chicago, USA). All experiments were performed in triplicate. *P* < 0.05 was considered statistically significant.

## Results

### miR-10b was down-regulated and HOXA3 and YAP were up-regulated in ccRCC cells

**Fig. 1:**
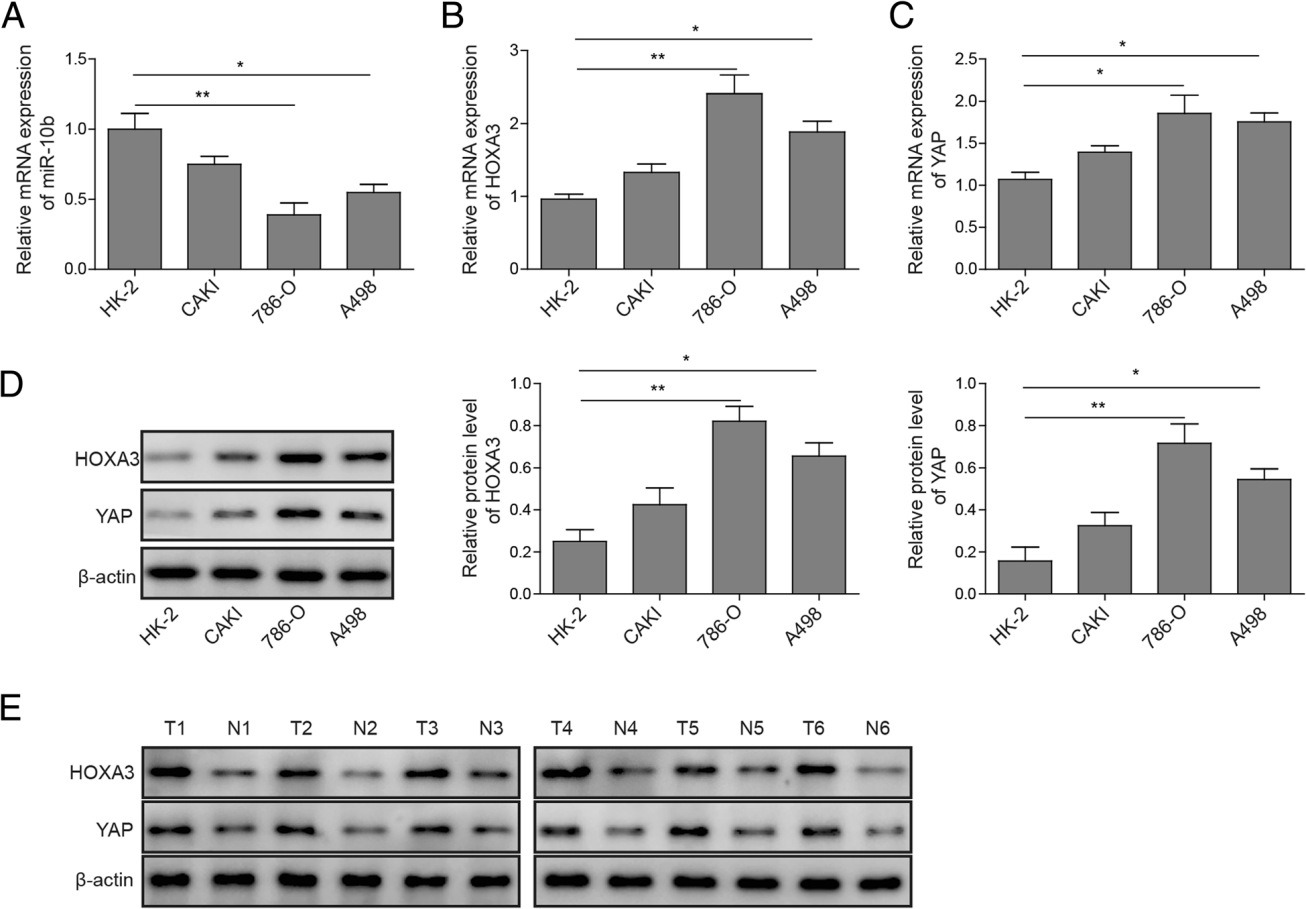
Expressions of miR-10a, HOXA3 and YAP in ccRCC cell lines. qRT-PCR was performed to determine the mRNA expression of (**a**) miR-10b, (**b**) HOXA3, and (**c**) YAP in human ccRCC 786-O, and A498 cells, renal tubular HK-2 cells, and non-VHL mutated cancer CAKI cells. **d** Western blot was performed to determine HOXA3 and YAP protein expressions in 786-O, A498, CAKI and HE-2 cells; β-actin was used as a control. **e** Western blot was performed to determine HOXA3 and YAP protein expressions in 6 fresh human ccRCC tissues and the paired adjacent normal tissue samples. *P < 0.05, **P < 0.01, compared to cells treated with control

Given that miR-10b is a negative regulator of ccRCC cell metastasis, we first detected the miR-10b expression level in ccRCC cell lines, CAKI cells, as well as HE-2 cells. As presented in Fig. 1a, the level of miR-10b mRNA was markedly downregulated in both 786-O and A-498 cells, and to a lesser degree, in CAKI cells, as compared to HE-2 cells. We next examined the expression of HOXA3 and YAP in ccRCC. HOXA3 and YAP mRNA expression was markedly upregulated in ccRCC cells, and to a lesser extent in CAKI cells, as compared to HE-2 cells (Fig. 1b and c). Western blot analysis also demonstrated that the ccRCC cell lines exhibited stronger signals of HOXA3 and YAP than CAKI or HE-2 cells (Fig. 1d). Moreover, HOXA3 and YAP were significantly upregulated in 6 fresh human ccRCC tissues, compared with that in adjacent normal tissue samples (Fig. 1e). These data suggest the involvements of miR-10, HOXA3 and YAP in ccRCC.

### Knockdown of YAP inhibits proliferation, migration and invasion of ccRCC cells

**Fig. 2:**
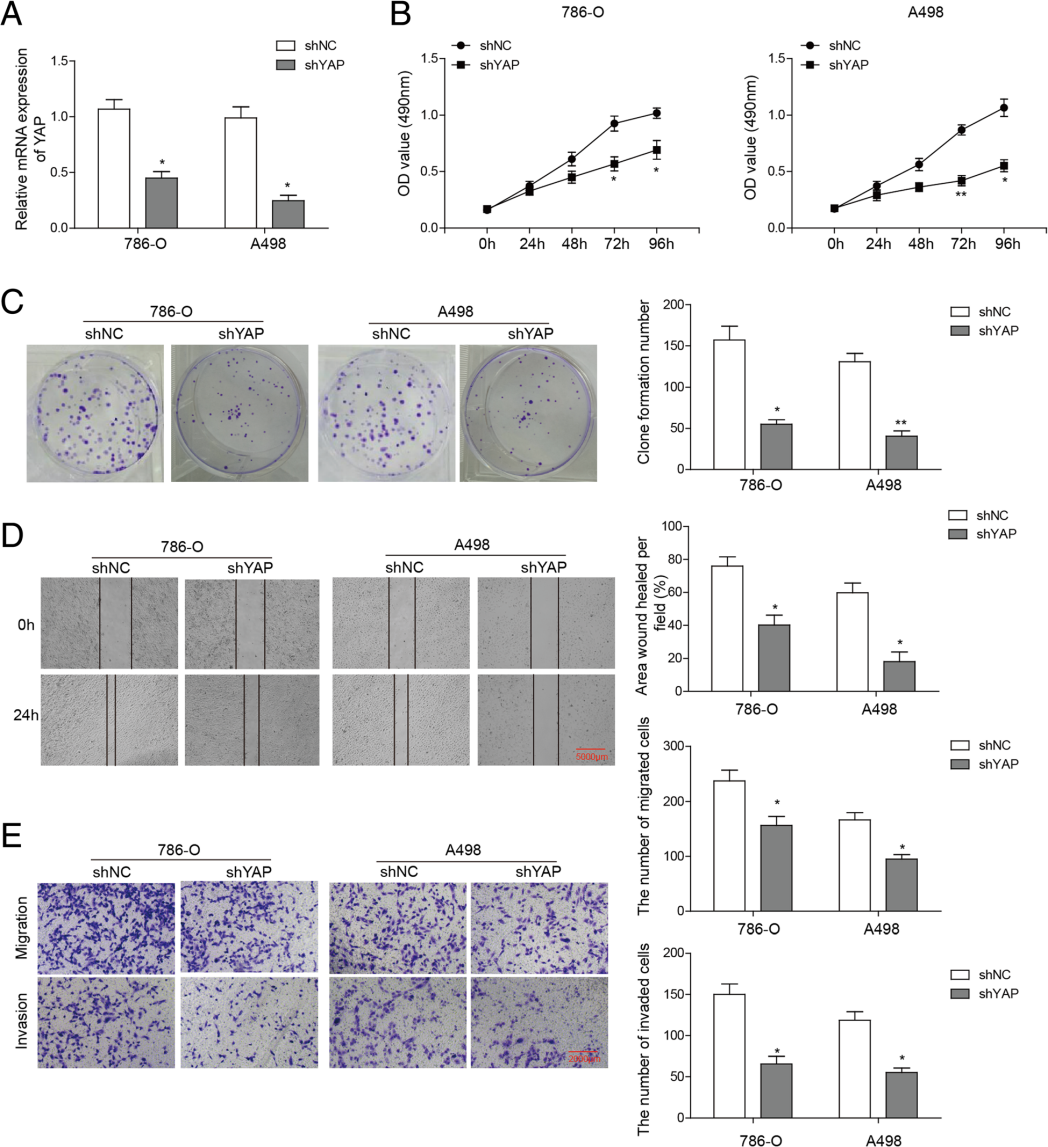
Effect of YAP knockdown on cell growth, colony formation, migration and invasion of RCC cells. **a** qRT-PCR showed a marked reduction of YAP expression in 786-O and A498 cells with YAP knockdown. **b** MTT assay showed that knockdown of YAP in 786-O and A498 cells significantly reduced cell viability compared with the parental cells. **c** Colony formation assay showed that 786-O and A498 cells with YAP knockdown formed fewer colonies compared with the parental cells. **d** Wound-healing assay. Cells were quantified by counting three separate fields in different cells. The migratory ability of 786-O and A498 cells with YAP knockdown were remarkably decreased than those of parental cells. **e** Transwell assay. The invasion and migration number of cells in 786-O and A498 cells with YAP knockdown were lower than those in the parental cells. *P < 0.05, **P < 0.01, compared to cells treated with control

We further examined the function of YAP in proliferation, migration and invasion of ccRCC cells. qRT-PCR reveled a marked reduction of YAP level in 786-O and A498 cells after YAP knockdown (Fig. 2a) and MTT assay showed clear inhibition of cell viability (Fig. 2b). Furthermore, YAP knockdown suppressed colony formation, decelerated the wound closure rate and reduced migration and invasiveness of ccRCC cells, as detected by colony formation assay, wound-healing assay and transwell assay, respectively (Fig. 2c-e). These various observations suggest that knockdown of YAP could inhibit proliferation, migration and invasion of ccRCC cells.

### Knockdown of YAP down-regulates HOXA3 and MMP-9 gene expression in ccRCC cells

**Fig. 3:**
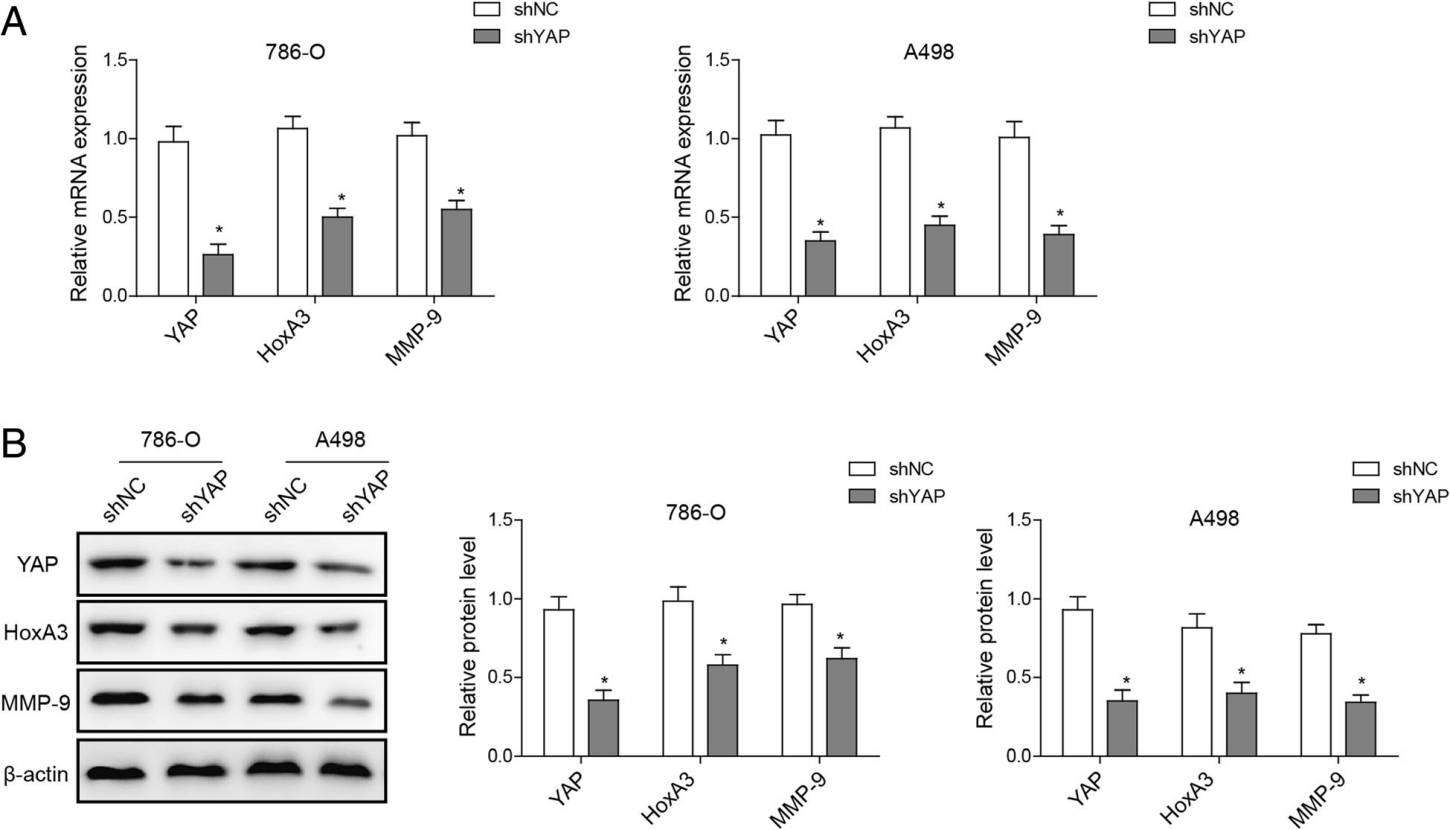
Knockdown of YAP down-regulates HOXA3 and MMP-9 gene expression in ccRCC cells. **a** qRT-PCR showed a marked reduction in the mRNA expression of YAP, HOXA3 and MMP-9 in 786-O and A498 cells with YAP knockdown. **b** Western blot was performed to determine YAP, HOXA3 and MMP-9 expressions in 786-O and A498 cells with YAP knockdown; β-actin was used as a control. *P < 0.05, compared to cells treated with control

Apart from a marked reduction of YAP level in ccRCC cells with YAP knockdown, expression levels of HOXA3 and the metastasis-associated gene MMP-9 were also significantly reduced, as detected by qRT-PCR (Fig. 3a). Western blotting analyses showed similar results (Fig. 3b). These data suggest a mechanism by which YAP may potently up-regulate HOXA3 expression and other genes required for cell metastasis.

### Knockdown of FAK down-regulates YAP, HOXA3 and MMP-9 gene expression in ccRCC cells

**Fig. 4:**
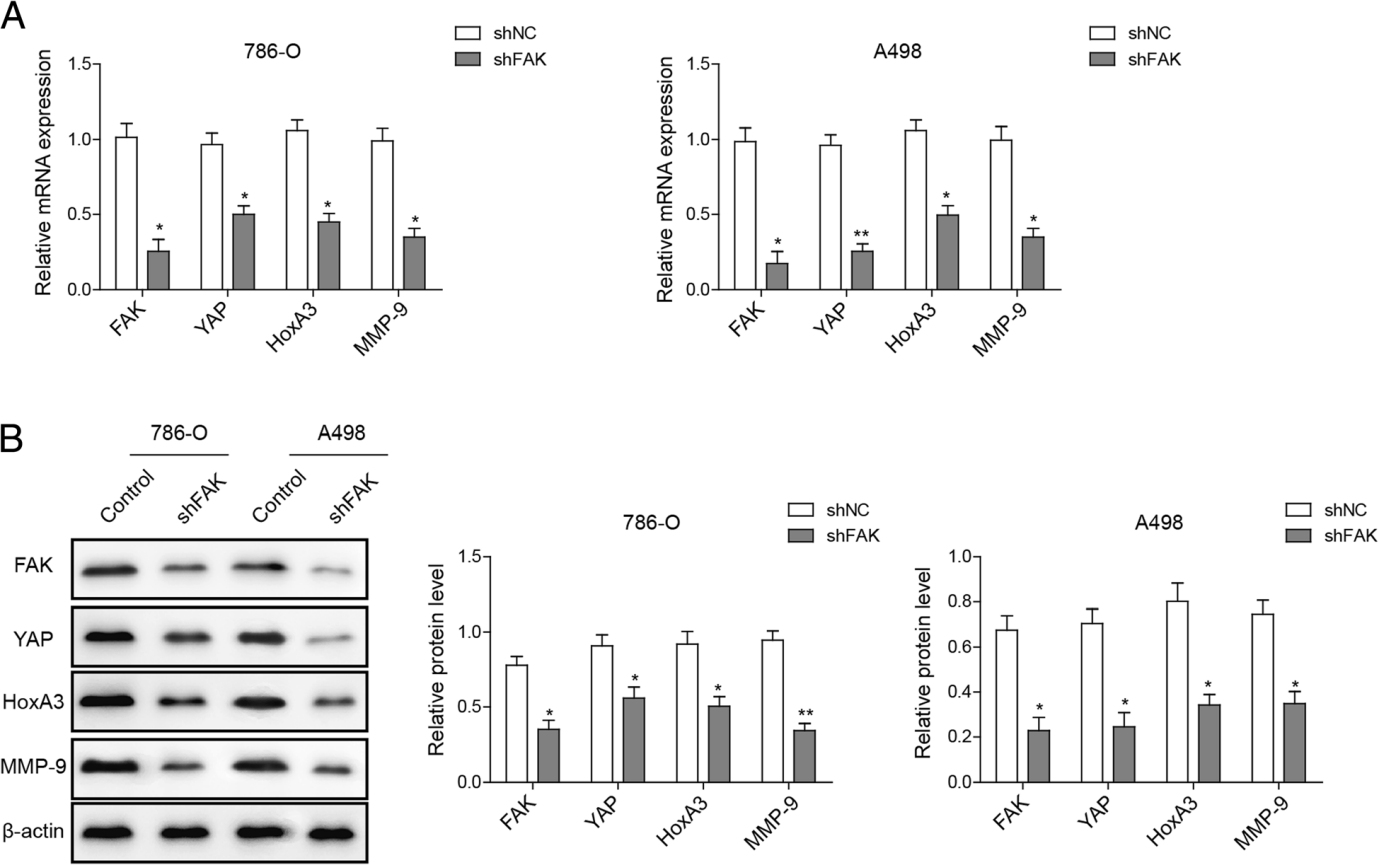
Knockdown of FAK down-regulates YAP, HOXA3 and MMP-9 gene expression in ccRCC cells. **a** qRT-PCR showed a marked reduction in the mRNA expression of FAK, YAP, HOXA3 and MMP-9 in 786-O and A498 cells with YAP knockdown. **b** Western blot was performed to determine FAK, YAP, HOXA3 and MMP-9 expressions in 786-O and A498 cells with YAP knockdown; β-actin was used as a control. *P < 0.05, **P < 0.01, ***P < 0.001, compared to cells treated with control

To investigate the influence of FAK activity on YAP activation in ccRCC cell lines, we used shRNA to knock down the endogenous expression of FAK in 786-O and A498 cells, and observed a significant decrease in FAK expression in ccRCC cells with FAK shRNA. Consistent with this, the mRNA expression of the YAP and its potential target genes HOXA3 and MMP-9 were also significantly downregulated (Fig. 4a). This decrease in expressions was independently confirmed by Western blot analysis (Fig. 4b). Collectively, our data indicate that FAK inactivation represses the YAP expression, which further down-regulates HOXA3 and MMP-9 in ccRCC cells.

### HOXA3 is a direct target of miR-10b, which suppresses proliferation and invasion capabilities in ccRCC cells

**Fig. 5:**
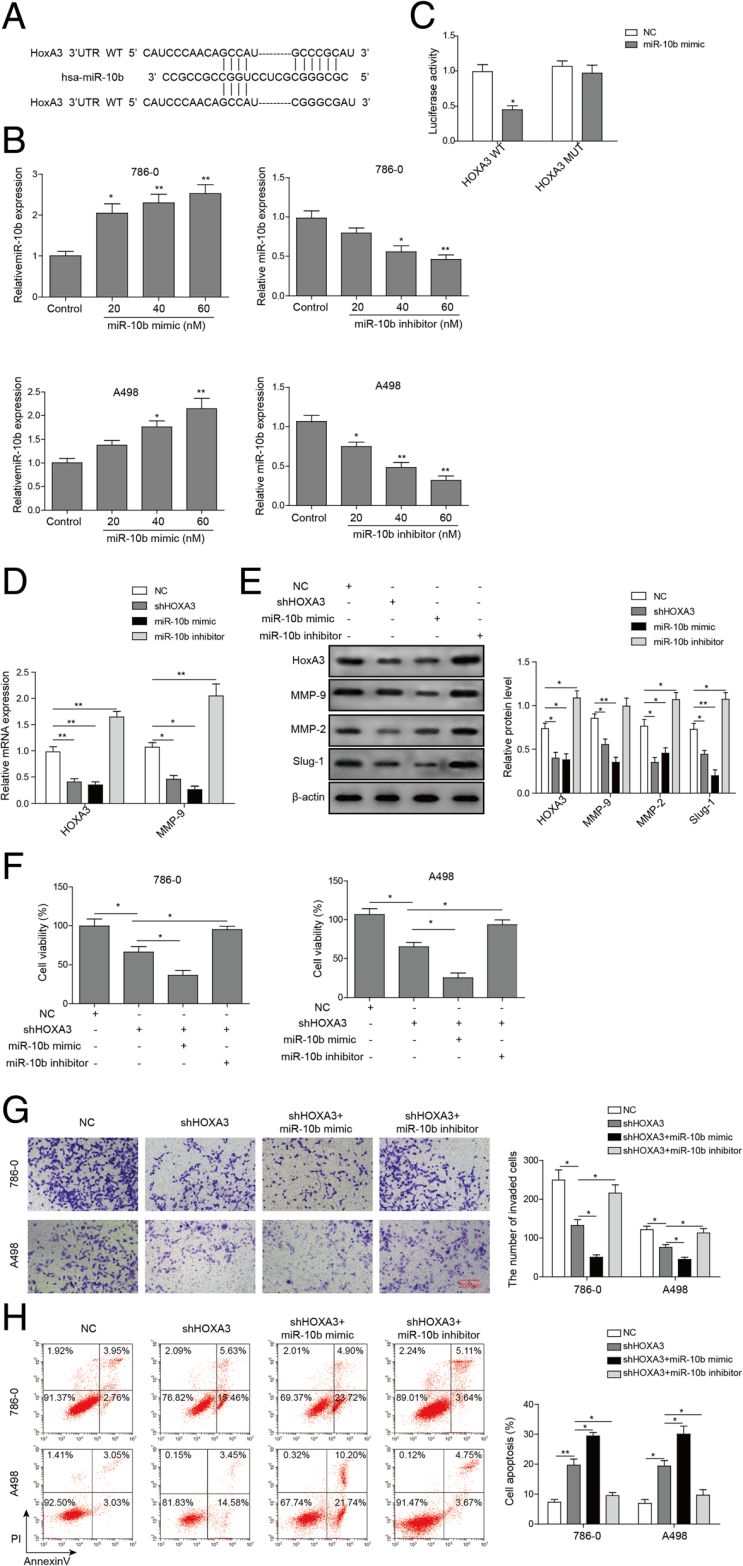
HOXA3 functioned as a target of miR-10b, which suppressed proliferation, invasion and apoptosis of ccRCC cells. **a** Predicted duplex formation between human HOXA3 3’UTR and miR-10b. **b** The relative levels of miR-10 in ccRCC cells transfected with miR-10b mimic and miR-10b inhibitor, as determined by qRT-PCR. RNU44 was used as endogenous control. **c** Luciferase activity of wild-type (UTR-wt) or mutant (UTR-mut). HOXA3 3’UTR reporter gene in 786-O cells infected with the miR-10b mimic or negative control (NC) mimic. **d** qRT-PCR of HOXA3 in 786-O cells infected with shHOXA3, miR-10b mimic, miR-10b inhibitor or vector. **e** Western blot of HOXA3 in 786-O cells infected with shHOXA3, miR-10b mimic, miR-10b inhibitor or vector. **f** Cell viability of 786-O and A498 cells transfected with negative control (NC), shHOXA3, shHOXA3 + miR-10b mimic or shHOXA3 + miR-10b inhibitor for 48 h, as determined by MTT assay. **g** Invasion of ccRCC cells transfected with negative control (NC), shHOXA3, shHOXA3+ miR-10b mimic or shHOXA3 + miR-10b inhibitor, as gauged by transwell invasion assay. **h** Apoptosis of ccRCC cells transfected with negative control (NC), shHOXA3, shHOXA3+ miR-10b mimic or shHOXA3 + miR-10b inhibitor. *P < 0.05, **P < 0.01, compared to cells treated with control

To further explore the mechanisms underlying the inhibitory effects of miR-10b on tumor invasion and metastasis, we performed bioinformatics analysis to identify the potential of HOXA3 as one target gene of miR-10b in ccRCC cells. The results revealed that the HOXA3-encoded mRNA contains a 3’UTR element that is complementary to miR-10b and has identical sequence in the human mRNA orthologues (Fig. 5a). Furthermore, we transfected 786-O and A498 cells with 0, 20, 40, 60 nM miR-10b mimic and inhibitor, and found that a dose-dependent increase and decrease in miR-10b mRNA expression, respectively (Fig. 5b). We subsequently chose 40 nm miR-10b mimic or inhibitor for further experiments. We found that the miR-10b overexpression reduced 55% luciferase activity in cells transfected with wide-type HOXA3 3’UTR, while no apparent effect was observed on the luciferase activity in cells transfected with mutant HOXA3 3’UTR, as determined by dual luciferase reporter assay (Fig. 5c). The data suggest that miR-10b targets HOXA3 and its function on HOXA3 depends upon the presence of a single miR-10b cognate binding site within the 3’UTR.

In support of these results, we found an apparent decrease in HOXA3 protein expression in cells transfected with the miR-10b mimic, to an extent comparable to that achieved by transfected the cells with shHOXA3. By contrast, a markedly increase in the level of HOXA3 protein was found in 786-O cells transfected with the miR-10b inhibitor. Similar results were also found for known metastasis-associated genes including MMP-2, MMP-9 and Slug-1(Fig. 5d and e). Collectively, our data indicate that HOXA3 is a direct and functional target of miR-10b in ccRCC cells.

We finally investigated the functional roles of miR-10b and shHOXA3 in ccRCC cells. MTT assays showed that HOXA3 knockdown and miR-10b enhancement significantly repressed cell viability in 796-O and A498 cells, while inhibition of miR-10b effectively reversed the shHOXA3-mediated cell activities (Fig. 5f). Furthermore, HOXA3 knockdown significantly inhibited cell invasion and promoted cell apoptosis, while concomitant administration with miR-10b mimic had a pronounced synergistic effect (Fig. 5g and h). Similarly, miR-10b depletion counteracted the tumor-suppressive activity of shHOXA3 (Fig. 5g and h). Taken together, miR-10b, which targeted HOXA3, could suppress proliferation and invasion in ccRCC cells.

## Discussion

MicroRNAs have been associated with tumor progression in various cancers including RCC [11–18], which is one of the most common malignant tumors [15–18]. Previous study has suggested the important regulatory function of miR-10b in renal cancer [18]. However, the direct target of miR-10b in renal cancer cell and related molecular mechanisms has not yet been revealed. In this study, we confirmed the crucial role of miR-10b in ccRCC cell proliferation, migration and invasion and demonstrated that miR-10b suppresses cell invasion and metastasis through targeting HOXA3 regulated by FAK/YAP signaling pathway in ccRCC.

Expression of miR-10b in tumor cells implied its essential role in ccRCC metastasis. In our functional validation study, miR-10b expressed a relatively low level in human renal tumor cell lines (Fig. 1). Furthermore, we demonstrated a correlation between up-regulation of miR-10b and the suppression of cell proliferation, migration and invasion in vitro, and a correlation of miR-10b inhibition with the reverse (Fig. 5). Although the gene expression profile of miR-10b in ccRCC is opposite to that found in most other human cancers in which miR-10b is generally considered to be a tumor suppressor [11–14], several previous studies on RCC, including the TCGA project, have demonstrated the down-regulation of miR-10b in RCC cells [15–18]. Possibly, the opposite functioning of this miR-10b in ccRCC could be explained by different cellular context of RCC molecular pathology [29].

The HOX3 gene belongs to HOX gene family that is important transcript factors regulating cell proliferation. HOX genes have been demonstrated to impact tumorigenesis directly via diverse mechanisms in cancer [19], and HOXB13 has been shown to play a critical regulatory role in renal cancer [22]. However, the function of HOXA3 in renal cancer is unclear. In our study, HOXA3 was found to be up-regulated in ccRCC cells (Fig. 1). Bioinformatics study suggested that HOXA3 mRNA is a potential target of miR-10b. To further confirm the direct targeting of miR-10b on HOXA3, we analyzed the correlation between miR-10b and HOXA3 through the design of miR-10b mimics and inhibitors to monitor the expression of miR-10b in renal cancer cell. Overexpressed miR-10b reduced HOXA3 expression to levels similar to those in ccRCC cells treated with shHOXA3, whereas miR-10b depletion dramatically enhanced HOXA3 expression (Fig. 5d and e). Moreover, concomitant treatment of shHOXA3 with miR-10 mimic exerted an obvious synergetic effect on tumor cell activity inhibition (Fig. 5f-h). In addition, inhibition of miR-10 could completely rescue the decreased proliferation, migration and invasion by HOXA3 knockdown. The results clearly showed that miR-10b regulates cell proliferation by targeting HOXA3 in renal cancer.

YAP is an effector of the evolutionarily conserved Hippo signaling pathway and plays key roles in regulating cellular proliferation, survival and differentiation [24–26]. YAP has been found to regulate the HOXA3 expression in oral and dental epithelial tissues and in the epidermis of skin during embryonic and adult stages [27], with FAK possibly controlling its nuclear translocation and activation [28]. However, whether YAP regulates the expression of HOXA3 in ccRCC and whether FAK is involved in this process have not been studied. In the current study, we demonstrated both the roles of YAP and FAK in the regulation of ccRCC metastasis and their correlations with HOXA3. In line with previous findings [30], knockdown of YAP dramatically inhibited proliferation, migration and invasion capabilities of ccRCC cells (Fig. 2). Moreover, YAP depletion resulted in a marked reduction in HOXA3 expression (Fig. 3), while FAK knockdown reduced both YAP and HOXA3 expression in ccRCC cells (Fig. 4). Apparently, these novel data suggest that YAP that is regulated by FAK, inhibits renal cell proliferation by downstream transcription factor HOXA3 in ccRCC. This FAK-YAP-HOXA3 pathway established in this study validates the regulatory roles of YAP and HOXA3 in renal cancer and how they were coordinated with the miR-10b to suppress the tumorigenesis of ccRCC.

In conclusion, our study demonstrated that miR-10b suppresses cell invasion and metastasis through targeting HOXA3, which partially passes through the FAK/YAP signaling pathway. Our findings provide insight into molecular mechanisms associated with the invasion of ccRCC cells and supply potential therapeutic targets for RCC metastasis.

## Abbreviations

ATCC: American Type Culture Collection
ccRCC: Clear-cell renal cell carcinoma
FAK: Focal adhesion kinase
FBS: Fetal bovine serum
HOX: Homeobox
miR-10b: microRNA-10b
YAP: Yes-associated protein

## References

[1] Siegel RL, Miller KD, Jemal A (2016). Cancer statistics, 2016. CA Cancer J Clin.

[2] Torre LA, Bray F, Siegel RL (2015). Global cancer statistics, 2012. CA Cancer J Clin.

[3] Moch H, Cubilla AL, Humphrey PA (2016). The 2016 WHO classification of tumours of the urinary system and male genital organs-part a: renal, penile, and testicular tumours. Eur Urol.

[4] De Meerleer G, Khoo V, Escudier B (2014). Radiotherapy for renal-cell carcinoma. Lancet Oncol.

[5] Karakiewicz PI, Briganti A, Chun FKH (2007). Multi-institutional validation of a new renal cancer-specic survival nomogram. J Clin Oncol.

[6] Albiges L, Fay AP, Xie W (2015). Efficacy of targeted therapies after PD-1/PD-L1 blockade in metastatic renal cell carcinoma. Eur J Cancer.

[7] Lamuraglia M, Rasian S, Elaidi R (2016). mTOR-inhibitor treatment of metastatic renal cell carcinoma: contribution of Choi and modified Choi criteria assessed in 2D or 3D to evaluate tumor response. Eur Radiol.

[8] Oudard S, Vano Y (2015). The role of rechallenge with targeted therapies in metastatic renal-cell carcinoma. Curr Opin Urol.

[9] Zanardi E, Verzoni E, Grassi P (2015). Clinical experience with temsirolimus in the treatment of advanced renal cell carcinoma. Ther Adv Urol.

[10] Zhang Y, Liao RB, Hu LL (2017). The microRNA miR-10b as a potentially promising biomarker to predict the prognosis of cancer patients: a meta-analysis. Oncotarget.

[11] Han X, Yan S, Weijie Z (2014). Critical role of miR-10b in transforming growth factor-beta1-induced epithelial-mesenchymal transition in breast cancer. Cancer Gene Ther.

[12] Bloomston M, Frankel WL, Petrocca F (2007). MicroRNA expression patterns to differentiate pancreatic adenocarcinoma from normal pancreas and chronic pancreatitis. JAMA.

[13] Ladeiro Y, Couchy G, Balabaud C (2008). MicroRNA profiling in hepatocellular tumors is associated with clinical features and oncogene/tumor suppressor gene mutations. Hepatology.

[14] Xiao H, Li H, Yu G (2014). MicroRNA-10b promotes migration and invasion through KLF4 and HOXD10 in human bladder cancer. Oncol Rep.

[15] Heinzelmann J, Henning B, Sanjmyatav J (2011). Specific miRNA signatures are associated with metastasis and poor prognosis in clear cell renal cell carcinoma. World J Urol.

[16] Slaby O, Redova M, Poprach A (2012). Identification of MicroRNAs associated with early relapse after nephrectomy in renal cell carcinoma patients. Genes Chromosomes Cancer.

[17] Osanto S, Qin Y, Buermans HP (2012). Genome-wide microRNA expression analysis of clear cell renal cell carcinoma by next generation deep sequencing. PLoS One.

[18] Fritz HK, Lindgren D, Ljungberg B (2014). The miR (21/10b) ratio as a prognostic marker in clear cell renal cell carcinoma. Eur J Cancer.

[19] Maeda K, Hamada J, Takahashi Y (2005). Altered expressions of HOX genes in human cutaneous malignant melanoma. Int J Cancer.

[20] Mace KA, Hansen SL, Myers C (2005). HOXA3 induces cell migration in endothelial and epithelial cells promoting angiogenesis and wound repair. J Cell Sci.

[21] Rauch T, Wang Z, Zhang X (2007). Homeobox gene methylation in lung cancer studied by genome-wide analysis with a microarray-based methylated CpG island recovery assay. Proc Natl Acad Sci U S A.

[22] Okuda H, Toyota M, Ishida W (2006). Epigenetic inactivation of the candidate tumor suppressor gene HOXB13 in human renal cell carcinoma. Oncogene.

[23] Khella HWZ, Daniel N, Youssef L (2017). miR-10b is a prognostic marker in clear cell renal cell carcinoma. J Clin Pathol.

[24] Zhang X, George J, Deb S (2011). The hippo pathway transcriptional co-activator, YAP, is an ovarian cancer oncogene. Oncogene.

[25] Overholtzer M, Zhang J, Smolen GA (2006). Transforming properties of YAP, a candidate oncogene on the chromosome 11q22 amplicon. Proc Natl Acad Sci U S A.

[26] Zender L, Spector MS, Xue W (2006). Identification and validation of oncogenes in liver cancer using an integrative oncogenomic approach. Cell.

[27] Liu M, Zhao S, Lin Q (2015). YAP regulates the expression of Hoxa1 and Hoxc13 in mouse and human oral and skin epithelial tissues. Mol Cell Biol.

[28] Lachowski D, Cortes E, Robinson B (2018). FAK controls the mechanical activation of YAP, a transcriptional regulator required for durotaxis. FASEB J.

[29] Gabriely G, Teplyuk NM, Krichevsky AM (2011). Context effect: microRNA-10b in cancer cell proliferation, spread and death. Autophagy.

[30] Chen KH, He J, Wang DL (2014). Methylationassociated inactivation of LATS1 and its effect on demethylation or overexpression on YAP and cell biological function in human renal cell carcinoma. Int J Oncol.

